# The Level of Circulating Endothelial Progenitor Cell Is Associated with Cerebral Vasoreactivity: A Pilot Study

**DOI:** 10.1155/2015/258279

**Published:** 2015-10-15

**Authors:** Chih-Ping Chung, Po-Hsun Huang, Jia-Shiong Chen, Jaw-Wen Chen, Kuang-Yao Yang

**Affiliations:** ^1^Department of Neurology, Taipei Veterans General Hospital, Taipei, Taiwan; ^2^National Yang Ming University, Taipei, Taiwan; ^3^Division of Cardiology, Department of Internal Medicine, Taipei Veterans General Hospital, Taipei, Taiwan; ^4^Department of Chest Medicine, Taipei Veterans General Hospital, Taipei 11217, Taiwan

## Abstract

Endothelial progenitor cell is known to be able to repair injured vessels. We assessed the hypothesis that endothelial progenitor cell also modulates cerebral endothelial function in healthy status. We used transcranial color-coded sonography to measure middle cerebral arterial vasoreactivity to CO_2_ (breath-holding index) in healthy subjects and observed its relationship with the number of circulating CD34CD133+ cells. To detect significant correlations between each characteristic and breath-holding index of middle cerebral artery, we used univariate and multivariate regression analyses. 22 young healthy subjects were included in the present study (6 men, 16 women; mean age: 28.45 ± 3.98 years, range: 22–34 years). The mean breath-holding index and CD45^low^CD34+CD133+ cells number were 0.95 ± 0.48% and 0.52 ± 0.26, respectively. The level of CD34CD133+ cells was independently associated with middle cerebral artery's vasoreactivity (*r* = 0.439, *P* = 0.04). Our results suggest that endothelial progenitor cell also modulates healthy cerebral vessels' endothelial function. This ability of endothelial progenitor cell could be potentially applied therapeutically and for prevention in conditions with cerebral endothelial dysfunction and cerebral ischemia.

## 1. Introduction

Circulating endothelial progenitor cell (CPC), mainly derived from the bone marrow, is identified by the surface expression of CD34 and CD133 epitopes (CD34+CD133+ cells) [1]. In response to ischemic stimuli, endothelial progenitor cell (EPC) will be mobilized from bone marrow to the ischemic sites with increased number of CPCs [2]. Increasing evidences have shown that EPC could contribute to, though to variant extents, reendothelialization and vasculogenesis after vascular damage [3–6]. Clinical studies have also proved the association of CPC with injured cerebral vessels [7–10]. Patients with a lower CPC number after acute ischemic stroke had a greater stroke severity and a poorer outcome [7–10]. Furthermore, numbers of CPCs have an inverse correlation with numbers of cerebral infarcts and a positive correlation with regional cerebral blood flow in patients with previous ischemic strokes [8].

Cerebral vascular endothelial function could be evaluated by cerebral vasoreactivity (CVR), which keeps cerebral blood flow (CBF) constant in response to physiological or pathological conditions. Although several vascular risk factors are associated with a decreased number of CPCs, in a study using flow-mediated brachial vasoreactivity measurement in healthy individuals, the numbers of CPCs were an independent and better predictor of vascular reactivity than the conventional vascular risk factors [11]. These results suggest that, under the status of endothelial injury by certain vascular risk factors, CPC might play a pivotal role in the vascular endothelial function repair. However, little is known about the role of CPC in cerebral endothelial function of healthy individuals without any vascular risk factors. We hypothesized that CPC is associated with cerebral endothelial function maintenance in healthy status without any evidence of vascular injury. CVR to CO_2_ is a surrogate of cerebral endothelial function [12]. In the present study, we used breath-holding test to measure CVR in healthy young subjects and to observe its relationship with the number of CPCs.

## 2. Materials and Methods

### 2.1. Subjects

Young healthy volunteers were recruited. People with hypertension, diabetes mellitus, heart diseases, dyslipidemia, and smoking history would be excluded. The Institutional Review Board of Taiwan Veterans General Hospital approved the study protocol and written informed consent was obtained from all the participants before recruiting into this study.

### 2.2. The Level of CPC

10 mL of peripheral blood samples was collected between 10.00 and 11.00 h, at least one hour after breakfast. Total mononuclear cells (MNCs) were isolated by density gradient centrifugation with Histopaque-1077 (density 1.077 g/mL, Sigma-Aldrich, St. Louis, MO). Isolated MNCs were then incubated with 10 *μ*L of FITC-conjugated anti-human CD34 mAb (Biolegend, San Diego, USA), 3 *μ*L of PE-conjugated anti-human mAb CD133 (Miltenyi Biotec Ltd., Surrey, UK), 10 *μ*L of PerCP-conjugated anti-human CD45 mAb (Becton Dickinson), and 10 *μ*L of APC-conjugated anti-human KDR mAb (R&D Systems Inc., Minneapolis, Minnesota, USA) at 4°C for 30 min prior to four-color flow cytometry analysis (FACScan, Becton Dickinson, Sunnyvale, California, USA). Control isotype immunoglobulin (Ig) G1 and IgG2a antibodies were obtained from Becton Dickinson. The CPCs were represented as CD45^low^CD34+CD133+ cells. The level of CPC was expressed as the percentage of CD45^low^CD34+CD133+ cell number/MNCs number (%). To assess the reproducibility of CPC measurements, CPCs were measured from 2 separate blood samples in 10 subjects, and there was a strong correlation between the 2 measurements (*r*  =  0.90, *P* < 0.001).

### 2.3. Transcranial Color-Coded Sonography

**Figure 1:**
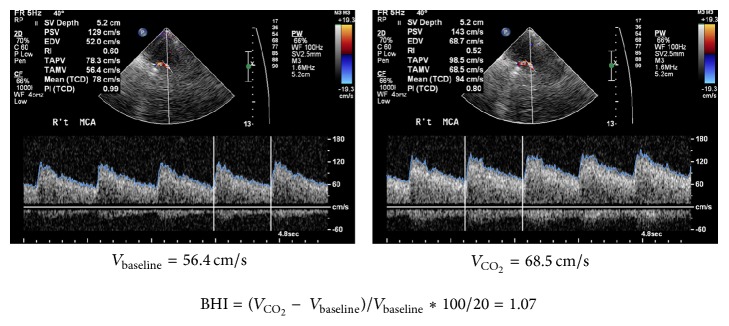
The breath-holing index (BHI) after 20 seconds of breath-holding calculated as percent increase in middle cerebral artery (MCA) mean blood velocity recorded by breath-holding divided by seconds of breath-holding ([*V*_CO_2__ − *V*_baseline_/*V*_baseline_]*∗*100/20).

To minimize the angle-corrected bias, we used transcranial color-coded sonography (TCCS) (iU22; Philips, New York, NY, USA), instead of transcranial Doppler (TCD), to evaluate the blood flow velocity in middle cerebral artery (MCA). TCCS was conducted by the same examiner using the Philips ultrasound system (iU22; Philips, New York, NY, USA). We used a center transmit frequency of 2 MHz in color mode, and Doppler gate at 5 to 10 mm. Angle-corrected velocity was determined whenever angle correction was less than 60 degrees in a straight arterial segment of at least 2 mm in length in all other cases. The waveform of the M1 segment of the MCA was determined through temporal skull window from depths of 45 to 65 mm as unidirectional signals toward the probe at the mesencephalic plane (Figure 1).

### 2.4. Breath-Holding Index (BHI)

All individuals performed the TCCS immediately after blood drawing and were lying for rest for at least 5 minutes before the TCCS examination. Firstly, the time-averaged-mean-velocity (TAMV) at baseline (*V*_baseline_) was obtained from the average of the mean blood flow velocity of three randomized cardiac cycles on Doppler spectrum. Individuals were then instructed to hold their breath after a normal expiration for 20 seconds. The TAMV of the last cardiac cycle at the end of breath-holding was recorded as the increased MCA blood flow in response to CO_2_ (*V*_CO_2__). TAMV acquisition was continuous from baseline to the end of breath-holding to make sure Doppler cursor was at the same position. TAMV measurements were made using built-in software (iU22; Philips, New York, NY, USA). The BHI was calculated from these data as percent increase in MCA mean blood velocity after breath-holding divided by seconds of breath-holding ([*V*_CO_2__ − *V*_baseline_/*V*_baseline_]*∗*100/20) (Figure 1) [13, 14]. In all individuals, we measured the BHI of MCA on the right side. Compared with transcranial Doppler, TCCS detects MCA flow velocity more reliably with visual support (color signals and anatomic associations with the other vasculatures and brain structures) and angle correction. In 10 subjects, the coefficient of variation of the measured BHI of MCA (an interval of 1 h) was 5%.

### 2.5. Statistical Analysis

Data were reported as the mean ± SD for numeric variables and as the number (percent) for categorical variables. Continuous variables were tested for normal distribution by the Kolmogorov-Smirnov test, and all variables passed it (*P* < 0.05). To detect significant correlations between each characteristic and BHI of MCA, we used univariate and multivariate regression analyses. The result of a correlation was represented by the correlation coefficient (*r*). It ranges from −1.0 to +1.0. The closer *r* is to +1 or −1, the more closely the two variables are related. If *r* is close to 0, it means that there is no relationship between the variables. For all tests, *P* < 0.05 was considered statistically significant. All analyses were performed with SAS software, version 9.1 (SAS Institute Inc., Cary, NC).

## 3. Results

Twenty-two healthy volunteers (6 men, 16 women; mean age: 28.45 ± 3.98 years, range: 22–34 years) were recruited. None had history of hypertension, diabetes mellitus, heart diseases, dyslipidemia, obesity, and smoking habits.

The clinical characteristics of the volunteers are demonstrated in Table 1. There are also results of univariate and multivariate regression analyses for detecting any possible correlation between the BHI of MCA and each characteristic. The results showed that age, gender, and physiological (BP, HR, and BMI) and metabolic (total cholesterol, HDL, LDL, fasting sugar, and CRP) values were not associated with BHI, respectively.

**Figure 2:**
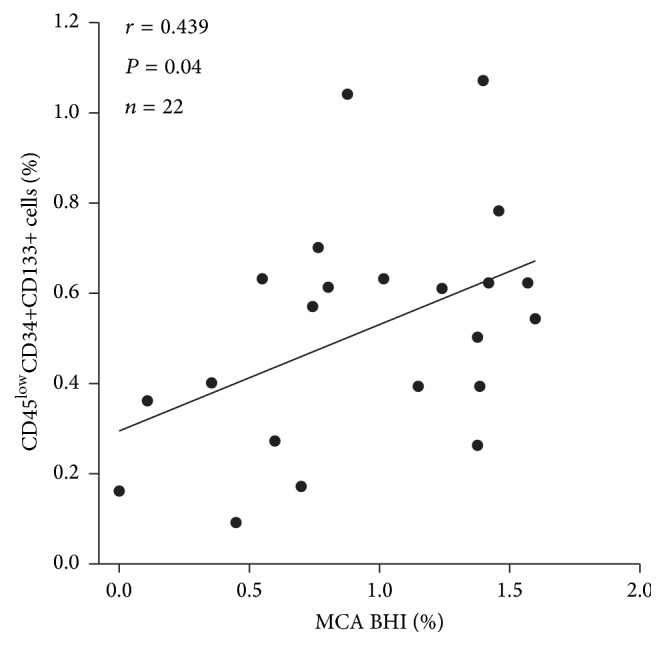
Relationship between breath-holding index (BHI) of middle cerebral artery (MCA) and the level of circulating endothelial progenitor cell (CPC).

Table 2 demonstrates the results of white blood cell profiles and sonographic data. Only the level of CPC had a significant effect on MCA's vasoreactivity response to CO_2_ in the multivariate analysis (*r* = 0.439, *P* = 0.04) (Figure 2).

## 4. Discussion

Current interest in EPCs centers predominantly on their role in angiogenesis and repairing of injured endothelium. We are the first to show that CPC is associated with cerebral endothelial function maintenance in healthy subjects. In our study, the level of CPC is the only predictor of CVR in healthy young individuals without any evidence of vascular injury.

The previous study has shown that the endothelial function assessed by flow-mediated dilatation of brachial artery was significantly associated with the number of EPC colonies in healthy middle-aged men [11]. The present study reveals that the number of CPCs is also associated with the cerebral vasoreactivity in the brain circulation. A lower level of CPCs has been found in (1) subjects with more severe age-related white matter changes [15], one kind of cerebral small vessel disease, and (2) ischemic stroke patients with unfavorable outcomes [7, 9, 10]. We postulate that a dysfunctional cerebral vasoreactivity may be involved in the pathophysiology.

The mechanism linking a lower level of CPCs and decreased cerebral vasoreactivity might be the paracrine effect of CPCs. Study has found that one of the CPC's functions is to promote cerebral endothelial function by increasing prostacyclin (PGI_2_) production and intracellular concentration of cAMP found in animals [16]. Otherwise, low levels of CPCs have been shown to be associated with higher levels of homocysteine [17]. Since homocysteine causes endothelial dysfunction [18], elevated levels of homocysteine may mediate the relationship between a lower level of CPCs and decreased cerebral vasoreactivity.

Our results suggest that CPC not only plays a critical role of vascular repair and promotes new vessels formation in ischemic tissues, but also modulates vasoreactivity in healthy vessels. This ability of CPC could be applied therapeutically to improve CBF through cerebral autoregulation enhancement in cerebral ischemia but not yet infarcted lesions (penumbra) or cerebral hypoperfusion status with poor cerebral autoregulation (e.g., leukoaraiosis; severe internal carotid artery stenosis).

There are limitations of the present study. It should be noted that this pilot study had a relatively small sample size and that future larger studies are needed to elucidate the relationship between CPC and cerebral autoregulation. In addition, another limitation of the study is related to its cross-sectional nature, and further longitudinal studies are needed to determine the temporal relationships. Besides, studies using another index of cerebral autoregulation other than BHI should be conducted to test our present results.

## 5. Conclusion

The results of this pilot study suggest that EPC modulates healthy cerebral vessels' endothelial function. This ability of EPC could be potentially applied therapeutically and for prevention in conditions with cerebral endothelial dysfunction and cerebral ischemia. Further studies on a larger population of healthy individuals and cerebrovascular diseases patients are needed.

## Figures and Tables

**Table 1:** Clinical characteristics of subjects and the correlations between each variable and middle cerebral artery breath-holding index.

	Subjects (*n* = 22)	*R*	*P* value
Age, years	28.45 ± 3.98	−0.081	0.719
Gender, M/F	6/16	−0.047	0.835
*Physiological values*			
Systolic BP, mmHg	111.32 ± 11.15	−0.019	0.933
Diastolic BP, mmHg	69.36 ± 7.13	−0.036	0.874
Heart rate, beats/min	75.86 ± 9.44	−0.075	0.741
Body mass index, kg/m^2^	21.34 ± 2.94	0.171	0.448
*Metabolic values*			
Total cholesterol, mg/dL	176.09 ± 15.63	−0.091	0.688
LDL-cholesterol, mg/dL	88.50 ± 15.96	−0.014	0.535
HDL-cholesterol, mg/dL	45.59 ± 15.56	−0.037	0.871
Fasting glucose, mg/dL	79.27 ± 11.25	0.189	0.400
C-reactive protein, mg/dL	0.05 ± 0.03	0.137	0.545

**Table 2:** White blood cell profile and sonographic parameters of subjects and the correlations between each variable and middle cerebral artery breath-holding index.

	Subjects (*n* = 22)	*R*	*P* value
*Blood cell levels*			
White blood cells, /*µ*L	6591 ± 957.63	0.017	0.940
Mononuclear cells, /*µ*L	525.45 ± 228.76	−0.177	0.432
CD45^low^CD34+CD133+ cells, %	0.52 ± 0.26	0.439	0.041^*∗*^
*Middle cerebral artery*			
*V*_baseline_, cm/s	80.40 ± 14.49	−0.383	0.078
*V*_CO_2__, cm/s	92.23 ± 20.59	−0.061	0.786
Breath-holding index, %	0.95 ± 0.48	—	—

^*∗*^Significant after multivariate analysis.
